# Efficient Biosynthesis of Acidic/Lactonic Sophorolipids and Their Application in the Remediation of Cyanobacterial Harmful Algal Blooms

**DOI:** 10.3390/ijms241512389

**Published:** 2023-08-03

**Authors:** Yuanyuan Xia, Yibo Shi, Jieyu Chu, Shiying Zhu, Xiaozhou Luo, Wei Shen, Xianzhong Chen

**Affiliations:** 1 Key Laboratory of Industrial Biotechnology, Ministry of Education, School of Biotechnology, Jiangnan University, 1800 Lihu Avenue, Wuxi 214122, China; yyxia@jiangnan.edu.cn (Y.X.); 7210201060@stu.jiangnan.edu.cn (Y.S.); 1023180311@stu.jiangnan.edu.cn (J.C.); 1023190101@stu.jiangnan.edu.cn (S.Z.); shenwei_micro@163.com (W.S.); 2 School of Biotechnology, Jiangnan University, Wuxi 214122, China; 3 Center for Synthetic Biochemistry, Shenzhen Institutes for Advanced Technologies, Chinese Academy of Sciences, Shenzhen 518055, China; xz.luo@siat.ac.cn

**Keywords:** sophorolipids, *Starmerella bombicola*, metabolic engineering, cyanobacteria degradation, cyanobacterial harmful algal blooms

## Abstract

Cyanobacterial harmful algal blooms (CyanoHABs) pose significant threats to human health and natural ecosystems worldwide, primarily caused by water eutrophication, increased surface water temperature, and co-occurring microorganisms. Urgent action is needed to develop an eco-friendly solution to effectively curb the proliferation of CyanoHABs. Sophorolipids (SLs) are fully biodegradable biosurfactants synthesized by *Starmerella bombicola*. They can be classified into lactone and acid types. The lactone type displays strong antimicrobial activity, while the acid type exhibits good solubility, which make them ideal agents for mitigating CyanoHABs. Nevertheless, the broad utilization of SLs are hindered by their expensive production costs and the absence of effective genetic editing tools in the native host. In this study, we constructed recombinant strains capable of producing either acidic or lactonic SLs using the CRISPR-Cas9 gene editing system. The yields of acidic and lactonic SLs reached 53.64 g/L and 45.32 g/L in a shaking flask, respectively. In a 5 L fermenter, acidic SLs reached 129.7 g/L using low-cost glucose and rapeseed oil as substrates. The addition of 5 mg/L lactonic SLs effectively degraded cyanobacteria within 30 min, and a ratio of 8.25:1.75 of lactonic to acidic SLs showed the highest degradation efficiency. This study offers a safe and promising solution for CyanoHABs treatment.

## 1. Introduction

Cyanobacteria are the most ancient phytoplanktons on the planet and form cyanobacterial harmful algal blooms (CyanoHABs) [1]. They are primarily caused by water eutrophication, increased surface water temperature, and co-occurring microorganisms [2,3,4]. In recent decades, CyanoHABs are geographically expanding across the globe, such as Erie, US–Canada; Lakes Victoria, Africa; Okeechobee, Florida, USA; Kasumigaura, Japan [5]; Taihu, China [6]; and the Baltic Sea in Northern Europe [7]. In the USA alone, CyanoHABs cause losses of over USD 2 billion of recreational, agricultural, and drinking water resources each year [8]. In 2007, a massive “cyanobacteria mat” in Lake Taihu caused considerable concern regarding drinking water safety and received significant media attention [9]. At present, the solutions mainly include manual salvage, ultrasonic waves, water diversion, and dilution [10]. Chemical agents such as triosyn, hydrogen peroxide, and copper sulfate were found competent in a small period of application [11]. In recent years, studies have reported that certain natural products can efficiently degrade cyanobacteria, such as cercosporin and sophorolipids (SLs) [12,13,14]. Compared with other agents, SLs exhibit remarkable advantages in dealing with large-scale CyanoHABs. The microbicidal activity of SLs is attributed to their membrane-perturbing properties, which rely on their partitioning into cell membranes [15]. Being biodegradable and environmentally friendly, they are ideal reagents for the green control of CyanoHABs [13]. Lee et al. reported that the sophorolipid–yellow clay mixture effectively mitigates HABs with fewer negative effects on the pelagic ecosystem [16].

**Figure 1:**
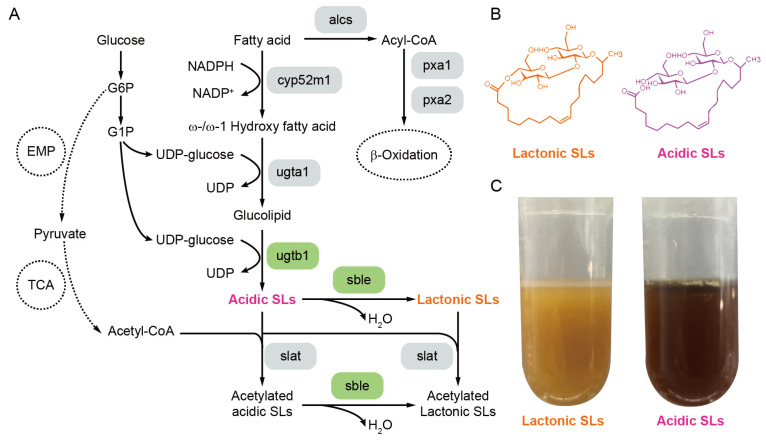
Synthetic metabolic pathway of SLs (**A**) and structural formulas of lactonic and acidic SLs (**B**), along with purified samples (**C**).

SLs are a class of amphiphilic glycolipid biosurfactants composed of two glucose groups and one fatty acid chain [17]. They are widely used because of good foaming, solubility, and emulsifying and bacteriostatic properties. In addition to the general properties of conventional surfactants, SLs are non-toxic, 100% biodegradable, highly tolerated, and environmentally friendly [18]. It has been reported that several non-pathogenic yeast species can synthesize SLs through a green and environmentally friendly fermentation process using sugar and vegetable oil as carbon sources [19]. *Starmerella bombicola* has gained significant attention as a strain capable of high-level production of SLs [17,20,21]. The wild-type strain mainly uses fatty acids and glucose as substrates to metabolize and synthesize SLs (Figure 1A), and the main metabolism process is that the ω-oxidation of fatty acids was catalyzed by cytochrome oxidase CYP52M1 to produce ω or ω-1 hydroxy fatty acids, followed by the catalysis of glucosyltransferase UGTA1; UDP-glucose is transferred to the hydroxyl position of hydroxy fatty acid to form glucose ester; then under the catalysis of glucosyltransferase UGTB1, another glucose group is added to form acidic SLs (Figure 1B). The acidic SLs can be further transformed into mono-acetyl or di-acetyl-type SL by acylase and then transferred extracellularly by multidrug resistance protein. The acidic SLs can be catalyzed by lactonase SBLE to generate lactonic SLs (Figure 1B). Currently, there are still issues associated with the production of SLs using microorganisms. Firstly, the metabolites of the wild-type strain consist of a mixture of both acidic and lactonic SLs. Therefore, different fermentation batches may lead to significant variations in the ratio of acidic to lactonic SLs in the product, thereby making it challenging to control SLs’ purity. This also limits the scope of its applications since both SLs have different structures, biologic activities, physicochemical properties, and fields of application. Generally speaking, lactonic SLs have high lipophilicity and high antibacterial, bactericidal, and anti-tumor activities [22]; while the acidic SLs have improved solubility and frothing characteristics, making them valuable in bioremediation, food, and cosmetics [23]. Another issue with the production of SLs using microbial fermentation is that it significantly relies on the raw materials used during the fermentation process. Furthermore, the cost of high-yielding strains’ raw materials is also usually high. For instance, Pekin reported that using honey and corn oil as fermentation raw materials can result in a 400 g/L yield. However, the high cost of these raw materials remains a significant obstacle to SLs’ industrial production [17,24]. Thirdly, an efficient gene editing method for *S. bombicola* is not yet available, which makes it challenging to obtain recombinant strains through metabolic engineering. Moreover, there is little research available on the type of SLs that is most efficient in the degradation of cyanobacteria. Currently, SLs are mainly naturally produced by selected strains of *S. bombicola*, and there are no genetically engineered strains available for industrial production.

Our previous study developed the CRISPR-Cas9-based and CRISPR-Cas12a gene editing method for *S. bombicola*, which can efficiently edit single, double, and even three genes simultaneously [25,26]. Furthermore, we screened and identified nine endogenous promoters of *S. bombicola* by analyzing its genome to establish a promoter library of various strengths [26]. In this study, we modified the metabolic pathway of *S. bombicola* to synthesize acidic or lactonic SLs by knockout or overexpression of critical enzymes using CRISPR-Cas9 gene editing technology, respectively. The recombinant strains of microbial fermentation biosurfactants were further increased by fermenter expansion and cultivation, and then different types of microbial fermentation biosurfactants were separated and purified. Finally, the degradation effects of acidic and lactone-type SLs on cyanobacteria were tested, and the optimal ratio of acidic and lactonic SLs for degrading cyanobacteria was determined.

## 2. Results and Discussion

### 2.1. Recombinant Strains Construction for Acidic and Lactonic SLs Production

In order to obtain a strain capable of producing acidic or lactonic SLs, we genetically modified *S. bombicola* using the CRISPR-Cas9 system. Since lactonase SBLE can catalyze the synthesis of lactonic SLs from acidic SLs, we knocked out the *SBLE* gene in the recombinant strain that had already knocked out the peroxisome ABC transporter-encoding gene *PXA1* in a previous study. Additionally, since UDP-glucose is transferred to the hydroxyl position of fatty acids to form glucose esters under the catalysis of glucose transferase UGTA1, and another glucose is added to glucose esters to form acidic SLs under the catalysis of glucose transferase UGTB1, we integrated the *UGTB1* gene at the deleted *SBLE* gene locus and replaced the strong promoter PTEF1 to enhance the synthesis of acidic SLs. The resulting strain capable of producing acidic SLs was named AciSb. To construct a strain capable of producing lactonic SLs, we used the same recombinant strain that had knocked out PXA1 as a basis and replaced the *SBLE* gene promoter with a stronger P_PGKI_ promoter in order to enhance the conversion of acidic SLs to lactonic SLs. The resulting strain capable of producing lactonic SLs was named LacSb.

### 2.2. Fermentation of AciSb and LacSb Strains

**Figure 2:**
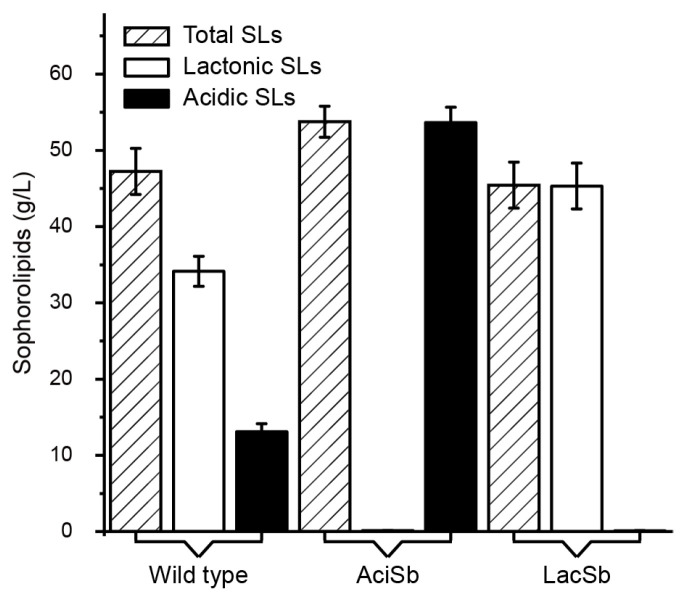
Determination of acidic and lactonic SLs of recombinant strains.

After successfully constructing AciSb and LacSb strains, we conducted fermentation verification using glucose as the carbon source and supplemented with rapeseed oil as the fatty acid source for shake-flask fermentation. Through purification, it can be observed that lactonic SL appears orange, while acidic SL appears brown (Figure 1C). The test results showed that both acidic and lactonic SLs were synthesized in the wild-type strain, with a total SL content of 47.24 g/L, including 34.15 g/L of acidic SLs and 13.09 g/L of lactonic SLs (Figure 2). As for the AciSb strain, the yield of acidic SLs reached 53.64 g/L, while almost no lactonic SLs were detected. In the case of the LacSb strain, the yield of lactonic SLs reached 45.32 g/L, while almost no acidic SLs were detected (Figure 2). Compared with the wild-type recombinant strain, the total SL content in the AciSb strain increased by 13.5%, and the acidic SL synthesized in the AciSb strain showed a 57% increase compared with the wild-type strain.

### 2.3. Fermentation of AciSb Strain in Fermenter

**Figure 3:**
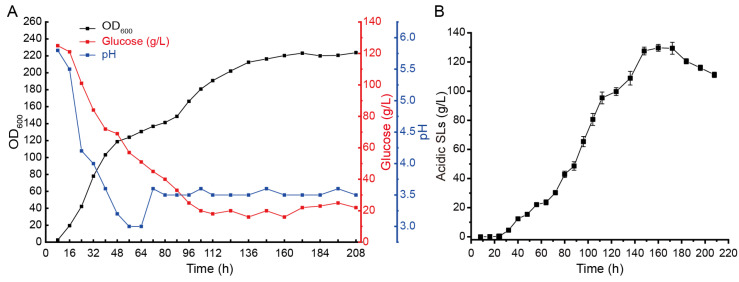
Fermentation of the AciSb strain in 5 L fermenter. Measurement of recombinant strain growth OD_600_, glucose consumption, and pH values (**A**). Quantification of acidic SL production (**B**).

Due to the higher sialic acid glycolipid synthesis ability of the acid-producing strain compared with the wild-type strain, we selected the acid-producing strain for scale-up fermentation in a 5 L fermenter to determine its ability to synthesize acid-type sialic acid glycolipids. The acid-producing strain was cultured overnight in a 50 mL YPD medium and then inoculated with a 5% inoculum into a 5 L fermenter containing a fermentation medium. The total volume of the fermentation broth was 3 L, and after 24 h of cultivation, rapeseed oil was added. As shown in Figure 3A, the strain rapidly grew before 48 h and then steadily increased until 172 h, with the OD_600_ reaching around 220. Looking at the glucose consumption rate, glucose was gradually consumed before 112 h. Additionally, during the early stage of cell growth, the pH of the fermentation broth decreased from the initial 6.0 to around 3.0. Later, the pH was stabilized at around 3.5 by adding ammonia solution. After 208 h of cultivation, the fermentation was terminated, and the highest yield of acid-type sialic acid glycolipids was obtained at 160 h of fermentation, reaching 129.7 g/L (Figure 3B). In our previous reports, the acidic SLs titer reached 99.5 g/L in a 5 L fermenter [26]. In this study, the production of acidic SLs can be further improved by enhancing the expression of UGTB1.

### 2.4. Study on the Degradation of Lactonic and Acidic SLs on Cyanobacteria

**Figure 4:**
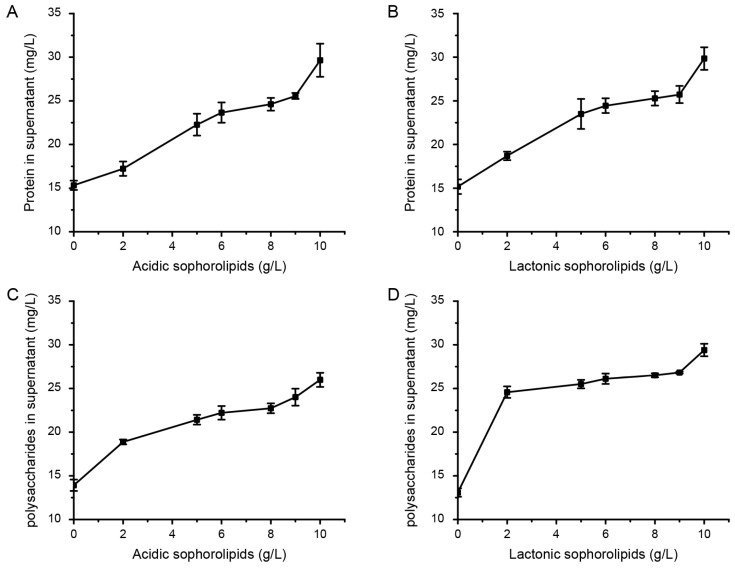
Determination of protein (**A**,**B**) and polysaccharide content (**C**,**D**) in the supernatant of cyanobacteria treated with different concentrations of acidic or lactonic SLs.

**Figure 5:**
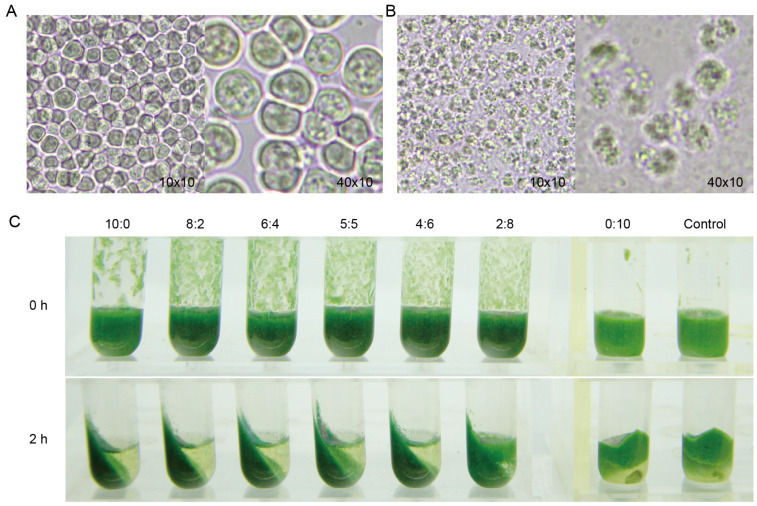
Observation of cyanobacterial cell morphology under an optical microscope at 100× and 400× magnification before (**A**) and after treatment (**B**) with 5 mg/L lactonic SLs for 30 min. Observation of cyanobacteria before and after treatment with different ratios of lactonic and acidic SLs through centrifugation (**C**).

The fermentation of lactonic and acidic SLs was carried out using AciSb and LacSb strains. After culturing, the culture medium was collected and purified to obtain pure acidic and lactonic SLs. The acidic SLs appeared coffee-colored, while the lactonic SLs appeared orange (Figure 1C). First, to determine whether the acidic or lactonic SLs have the ability to degrade cyanobacteria, we measured the protein and polysaccharide content in the solution after treating an equal amount of cyanobacteria with different concentrations of acidic or lactonic SLs. The results showed that as the concentration of acidic or lactonic SLs increased, the content of proteins and polysaccharides in the solution gradually increased, indicating that higher concentrations of SLs resulted in better degradation effects (Figure 4). Microscopic observation revealed that the untreated cells maintained their intact cellular morphology (Figure 5A), while the cells treated with 5 mg/L of lactonic SLs for 30 min appeared fragmented (Figure 5B).

### 2.5. Determination of Optimal Ratio of Lactonic/Acidic SLs for Cyanobacteria Degradation

**Figure 6:**
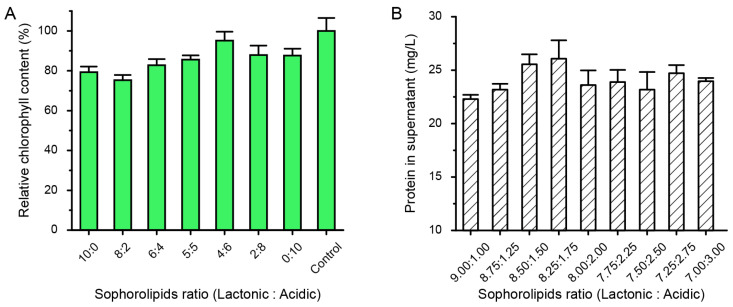
Quantification of relative chlorophyll content within cyanobacterial cells (**A**) and protein content in the supernatant (**B**) after treatment with different ratios of lactonic and acidic SLs.

To explore the degradation ability of mixed solutions of lactonic and acidic SLs on cyanobacteria, we mixed lactonic and acidic SLs in different ratios, allowed them to settle for 30 min, and then observed the degradation of cyanobacteria through centrifugation. Since live cyanobacterial cells float on the surface of the liquid after centrifugation, while degraded cyanobacterial cell fragments are present in the sediment, the centrifugation observation method can roughly determine the degradation effects of different ratios of lactonic/acidic SLs on cyanobacteria. The results showed that compared with the control, which was cyanobacteria treated with an equal amount of deionized water, the samples with different ratios of SLs exhibited some degree of degradation of cyanobacteria. The lactonic SLs have better degradation effect than acidic SLs. Among them, when the ratio of lactonic to acidic SLs was 8:2, the sediment of cells was the most significant, while there were almost no cyanobacteria on the liquid surface (Figure 5C). To more accurately explore the degradation ability of different ratios of lactonic/acidic SLs on cyanobacteria, we also measured the relative chlorophyll content in the treated cells. The results showed that when the ratio of lactonic to acidic SLs was 8:2, the residual chlorophyll in the cells was the lowest, indicating cell lysis and the release of chlorophyll into the solution, resulting in the most pronounced degradation effect (Figure 6A). To further determine the optimal ratio, we refined the ratios of lactonic/acidic SLs and determined the more precise ratio by measuring the protein content in the solution. The results showed that when the ratio of lactonic to acidic SLs was 8.25:1.75, the protein content in the solution was the highest, indicating the best degradation effect (Figure 6B). Although there are reports on the mitigation of harmful algal blooms using SLs [16], there is currently no report on the use of lactone-type or acid-type SLs and exploration of the effects of different mixing ratios on cyanobacterial degradation. From the results above, the lactonic SLs seem to exhibit better antimicrobial activities than acidic SLs.

## 3. Materials and Methods

### 3.1. Recombinant Strain Construction

The strains and primers used in this study are listed in Table 1 and Table 2, and the genes and primers were synthesized by GENEWIZ (Suzhou, China). The recombinant strain was constructed using the CRISPR-Cas9 system, and the detailed methods referred to a previous study [26]. For the recombinant strain AciSb, the construction of the gene integration cassette is as follows. The parental strain was previously constructed with the integrated Cas9 gene and overexpression of the *CYP52M1* (cytochrome P450 oxidoreductase) gene and the *CPR* (NADPH cytochrome P450 reductase) gene at the PXA1 (encoding peroxisome membrane transporter) and SBLE (encoding lactonase) loci. With the genome of *S. bombicola* as a template and SBLE-F/SBLE-R as a primer, a 2.5 kb *SBLE* gene fragment was amplified by PCR and ligated with the vector pMD19-T (Simple) using a ClonExpress II One Step cloning kit (Vazyme, Nanjing, China). The recombinant plasmid Ts-*SBLE* was then transferred to the *E. coli* JM109 clone. The recombinant plasmid Ts-*SBLE* was used as a template, and RSBL-F/RSBL-R was used as a primer. Reverse PCR was carried out under the action of Ex Taq DNA polymerase to obtain a vector of *SBLE*-Ts-*SBLE’* with a length of 3.3 kb, and the native promoter of the lactamase gene *UGTB1* was replaced with a stronger promoter P_TEF1_ (encoding transcriptional elongation factor EF-1α, accession number: MG719756). A gene knockout and insertion cassette Ts-*SBLE-hphex-P_TEF1_-UGTB1-Tgki-SBLE’* was constructed. After transferring the linearized gene knockout cassette and sgRNA into the *SbCas9* integration host cell, we obtained the AciSb strain (*S. bombicola* ATCC 22214, Δ*PXA1*::*P_ENO_-CYP52M1-P_TEF1_-CPR-hph*, Δ*SBLE*::*P_TEF1_-UGTB1-Tgki*). For the recombinant strain LacSb (*S. bombicola* ATCC 22214, *ΔPXA1:: P_ENO_-CYP52M1-P_TEF1_-CPR-hph*, *P_PGKI_-SBLE-Tgki*), the promoter of *SBLE* was replaced with P_PGKI_. The gene manipulation steps are similar to the above.

### 3.2. Cultivation and Fermentation

The *S. bombicola* and recombinant strains were cultured in standard YPD (10 g/L yeast extract, 20 g/L glucose, 20 g/L peptone, and 2% agar for making an agar plate). For the construction of recombinant strains, the selective medium (YPD supplemented with hygromycin B at a concentration of 0.5 g/L) and rich YPD medium (20 g/L yeast extract, 40 g/L glucose, and 40 g/L peptone) were used. A transformant capable of growing on an antibiotic-free plate but not on a plate containing hygromycin was selected for further culture, and finally a recombinant strain that does not contain a hygromycin resistance screening marker was obtained. The fermentation culture media contained 90 g/L glucose, 4 g/L yeast extract, 4 g/L sodium citrate, 1.5 g/L NH_4_Cl, 1 g/L KH_2_PO_4_, 0.16 g/L K_2_HPO_4_, 0.7 g/L MgSO_4_·7H_2_O, 0.5 g/L NaCl, and 0.27 g/L CaCl_2_·2H_2_O, pH 5.0. After striking the bacterial strain, a single colony was selected from the plate and inoculated into 5 mL of the YPD liquid medium in a 50 mL shake-flask. The culture was incubated for 12–16 h under conditions of 30 °C and 200 r/min in a constant-temperature incubator shaker (Zhichu, Shanghai, China). Subsequently, the culture was inoculated at a 1% rate into 50 mL of a fermentation medium in a 500 mL baffled conical flask. After 48 h of cultivation, 70.0 g/L of rapeseed oil was added to the medium. The pH of the fermentation medium was adjusted to 3.5 using 5 mol/L NaOH every 24 h. Batch fermentation in a 5 L fermenter with feeding was carried out as follows: Inoculate the seed liquid with 10% (*v*/*v*) inoculum into a 5 L fermenter containing 2 L of a fermentation culture medium. The AciSb and LacSb strains were cultured under the same conditions: 30 °C, pH 3.5 (using NaOH), the aeration rate is 2–4 vvm, and the agitation speed is set at 400–800 rpm (with dissolved oxygen above 20%). When the glucose concentration falls below 5 g/L, the addition of 800 g/L glucose supplement begins to maintain the glucose concentration around 5 g/L. After 48 h of cultivation, 70.0 g/L of rapeseed oil was added to the medium. The OD_600_ and residual sugar content were measured every 24 h during the fermentation period. After 9 days of fermentation, a sample was taken and the SL titer was determined.

### 3.3. Determination of SLs

The total SL content in the fermentation product was determined using the anthrone–sulfuric acid assay. A volume of 500 μL of fermentation broth was mixed with 1 mL of ethanol, and the mixture was vigorously shaken and centrifuged at 12,000 r/min (13,800× *g*) for 9 min. Then, 20 μL of the supernatant was transferred to an EP tube and mixed with 980 μL of distilled water and 4 mL of anthrone reagent. The mixture was immediately placed on ice and heated for 8 min in a boiling water bath. After cooling on ice, the absorbance of the reaction mixture was measured at 620 nm using a spectrophotometer. A solution obtained by reacting distilled water with anthrone reagent was used as a control. Based on the ratio of molecular weights between SLs and glucose, i.e., 1.91 g of SLs is equivalent to 1 g of glucose, the total SL content can be calculated based on a glucose standard curve. For the measurement of the lactonic SL content, the fermentation broth can be extracted with ethyl acetate using a similar method. The acidic SL content is calculated as the difference between the total SL content and the lactonic SL content. Furthermore, the content of SLs was determined by gas chromatography–mass spectrometry (GC/MS). The purified SL was dissolved in a solution of 2 mL of 1% H_2_SO_4_ in methanol (*v*/*v*). To ensure accurate analysis, 1 mL of toluene was added as an internal standard. The mixture was then subjected to heating at 100 °C for 1 h. Following this, 5 mL of cyclohexane and 5 mL of a 50 g/L NaCl solution were introduced. After centrifugation, the cyclohexane phase containing the fatty acid ester was carefully transferred to a fresh Eppendorf tube for subsequent analysis using GC/MS (SCIONSQ-456-GC, Bruker Inc., Billerica, MA, USA) equipped with an RTX-5 fused silica column (30 m × 0.32 mm, 0.25 μm coating) [27]. Three biological replicates were performed for each sample.

### 3.4. Purification of SLs

To purify the acidic or lactonic SLs, the fermented broth was first centrifuged to collect the supernatant. Then, two times the volume of either anhydrous ethanol or ethyl acetate was added to the supernatant and vortexed. The mixture was then subjected to vacuum concentration at 55 °C. After completion of rotary evaporation, an equal volume of n-hexane was added and again rotary evaporated. The resulting product was dried overnight in an oven to obtain purified acid or lactonic SLs. The purity of SLs was determined by MALDI-TOF MS (Bruker Daltonics, Bremen, Germany).

### 3.5. Study of Cyanobacteria Degradation

The present study utilized fresh cyanobacteria obtained from Lake Taihu, China, and Microcystis and Anabaena are reported as the main cyanobacteria that cause CyanoHABs in Taihu Lake [28]. Firstly, dead cyanobacteria cells were removed from the precipitate through centrifugation, and the upper cyanobacteria cells were collected and resuspended in distilled water. Subsequently, the cyanobacteria’s optical density was measured at 665 nm using a microplate spectrophotometer system (Spectra max190-Molecular Devices) and standardized to OD_665_ of 0.5 with distilled water. Purified acidic and lactonic SLs were prepared in specific ratios and added to the cyanobacterial suspension. The mixture was allowed to settle at 25 °C and then centrifuged at 4000 r/min (1878*× g*). The supernatant and sediment were evaluated for their cell content to assess the apparent degradation effect. The protein, polysaccharide, and chlorophyll content of cyanobacteria cells treated with SLs were measured, and their morphology was observed with an upright fluorescent microscope (model 80i; Nikon, Tokyo, Japan).

The protein content in the mixture was determined using the Bradford method. An amount of 500 μL of the supernatant was mixed with 500 μL of Bradford solution, and the absorbance at 595 nm was measured after 10 min of reaction at room temperature. The protein concentration was calculated based on the BSA standard curve.

The polysaccharide content in the solution was determined as follows: 200 μL of the sample was mixed with 200 μL of 5% phenol and 700 μL of concentrated sulfuric acid, and the mixture was incubated at 80 °C for 10 min. The absorbance at 490 nm was measured, and the polysaccharide content was calculated based on the glucose standard curve.

Chlorophyll was detected as follows: the cyanobacteria were treated with SLs for 2 h, and the solution containing the cells was obtained by filtration; the filter paper with the cells was placed in a centrifuge tube [29]. Then, 8 mL of 90% acetone solution was added and left to stand in a dark refrigerator (−20 °C) for 12 h. After taking it out and returning to room temperature, it was centrifuged for 1 min, and the supernatant was collected. The absorbance at 663 nm and 645 nm was measured, and the chlorophyll content was calculated using the formula Ca = 10.115E_663_ − 1.877E_645_.

## 4. Conclusions

In conclusion, this article highlights the successful application of metabolic engineering techniques to engineer *S. bombicola* for the production of acidic and lactonic SLs. This study achieved the production of acidic SLs using glucose and rapeseed oil as the sole carbon sources in a 5 L fermenter. Additionally, the investigation determined the optimal ratio of lactone-type to acidic SLs for effective cyanobacterial degradation, addressing a research gap in the field. While SLs have shown potential for cyanobacterial control, their high fermentation costs have hindered large-scale implementation. Therefore, currently, SLs are primarily commercially utilized in higher-value-added detergent and cosmetic products. However, the high-yield strain and optimized fermentation process developed in this study offer significant cost reductions for sophorolipid production. It was reported that the cyanotoxin produced by *Microcystis* is β-cyclocitral, so the release of cyanotoxin is also a threat to water quality [30]. In reality, even without any lysing agents, the death of cyanobacterial cells can result in the release of cyanotoxin. Therefore, further research directions include exploring the combination of SLs with clay for cyanobacterial control in diverse water environments [31,32]. For example, mixing SLs with clay can help sediment the cells to the bottom of the water while lysing them, utilizing the sedimentation capacity of the riverbed to reduce the contamination of cyanotoxin in the water. Additionally, the timing of using SLs to control cyanobacteria is preferably selected during months when cyanobacterial reproduction is slower, allowing for their control before a significant increase in cell proliferation occurs. Future efforts will focus on increasing the yield of recombinant strains, fine-tuning fermentation processes to lower production costs, and investigating the synergistic effects of various additives in conjunction with SLs to enhance cyanobacterial degradation efficiency. Ultimately, the aim is to apply this technology on a large scale in real water bodies to mitigate the ecological damage caused by CyanoHABs and alleviate the associated water supply crises. The successful implementation of this research holds promise for reducing the detrimental effects of harmful algal blooms and safeguarding the well-being of local communities.

## Figures and Tables

**Table 1:** The strains used in this study.

Name	Genotype	Reference
Wild type	*S. bombicola* ATCC 22214	This study
AciSb	*S. bombicola* ATCC 22214, *ΔPXA1::P_ENO_-CYP52M1-P_TEF1_-CPR-hph, ΔSBLE::P_TEF1_-UGTB1-Tgki*	This study
LacSb	*S. bombicola* ATCC 22214, *ΔPXA1::P_ENO_-CYP52M1-P_TEF1_-CPR-hph, P_PGKI_-SBLE-Tgki*	This study

**Table 2:** The primers used in this study.

Primers	Sequences	Restriction Sites
PTEF1-F	tcgtacaagtggcttacaaaacgcgtgtcctatggcttctgctttg	*Mlu* I
PTEF1-R	actggtttctcgatggccatactagtttttcaaattaagttttttg	*Bam*H I
UGTB-F	caaaaaacttaatttgaaaaactagtatggccatcgagaaaccagt	
UGTB-R	ctcgcatgtatgcacgtctacccgggtttgaaaaaatttatttctagacagttatatattaagaagctaattcactaa	
SBLE-F	cgcgcggatcttccagagatgggcccattggaacctagcccataag	*Apa* I
SBLE-R	gcacgcctgccgttcgacgagggcccaaacctactgctctgccgat	*Apa* I
RSBL-F	cggggtacccctgagcacgtattccgcta	
RSBL-R	cgacgcgttttgtaagccacttgtacgac	

## Data Availability

The data that support the findings of our study are available from the corresponding author upon reasonable request.
